# Polarity protein Canoe mediates overproliferation via modulation of JNK, Ras‐MAPK and Hippo signalling

**DOI:** 10.1111/cpr.12529

**Published:** 2018-10-17

**Authors:** Zhiwei Ma, Ping Li, Xingjie Hu, Haiyun Song

**Affiliations:** ^1^ Shanghai Institute of Nutrition and Health, Shanghai Institutes for Biological Sciences University of Chinese Academy of Sciences, Chinese Academy of Sciences Shanghai China; ^2^ School of Public Health Guangzhou Medical University Guangdong China; ^3^ School of Public Health Shanghai Jiao Tong University School of Medicine Shanghai China

**Keywords:** Canoe/Afadin, Hippo signalling, JNK signalling, Ras signalling, tumorigenesis

## Abstract

**Objectives:**

Over the past decade an intriguing connection between cell polarity and tumorigenesis has emerged. Multiple core components of the junction complexes that help to form and maintain cell polarity display both pro‐ and anti‐tumorigenic functions in a context‐dependent manner, with the underlying mechanisms poorly understood.

**Materials and Methods:**

With transgenic fly lines that overexpress or knock down specific signalling components, we perform genetic analysis to investigate the precise role of the polarity protein Canoe (Cno) in tumorigenesis and the downstream pathways.

**Results:**

We show that overexpression of *cno* simultaneously activates JNK and Ras‐MEK‐ERK signalling, resulting in mixed phenotypes of both overproliferation and cell death in the *Drosophila* wing disc. Moderate alleviation of JNK activation eliminates the effect of Cno on cell death, leading to organ overgrowth and cell migration that mimic the formation and invasion of tumours. In addition, we find that the Hippo pathway acts downstream of JNK and Ras signalling to mediate the effect of Cno on cell proliferation.

**Conclusions:**

Our work reveals an oncogenic role of Cno and creates a new type of *Drosophila* tumour model for cancer research.

## 1 INTRODUCTION

Cell polarity is the morphological and functional asymmetry that helps to form tissue structures such as tubes and alveoli.^1^, ^2^ This type of asymmetric distribution of intracellular proteins is established and maintained mainly via cell‐cell junctions and adhesions that are formed by a diversity of polarity proteins and adhesion molecules.^3^, ^4^ Among these are three groups of core regulators of cell polarity, including the apical Crumbs polarity complex that consists of Crumbs (Crb)/Pals/Patj, the basolateral Par polarity complex that consists of Cdc42/Par3/Par6/aPKC and the Scribble polarity complex that consists of Scribble (Scrib)/Discs large (Dlg)/Lethal (2) giant larvae (Lgl).^5^, ^6^ Over the last decade, evidence has been accumulating for the link between tumorigenesis and defects in cell polarity. For instance, in mammary epithelial cells, depleting Crb or Pals caused dephosphorylation and nuclear translocation of YAP, which is a crucial step for the inhibition of the Hippo signalling pathway and results in increased cell proliferation.^7^, ^8^ In a DMBA/TPA‐induced skin cancer model, the loss of Par3, aPKC or both, strongly reduced tumour size and multiplicity, mainly via impairing the activation of ERK1/2 and Akt by Ras.^9^ In mammary epithelia, dysregulation of Scrib prevented Myc‐induced apoptosis and promoted epithelial‐mesenchymal transition (EMT) and tumorigenesis.^10^, ^11^ The effects of cell polarity disruption on tumour growth rely on the association and regulation of downstream signalling pathways.^12^, ^13^ Besides, the roles of polarity proteins in tumorigenesis seem complicated, displaying either pro‐ or anti‐tumorigenic functions and largely depending on the context of the cells.^5^, ^13^, ^14^

The adhesion molecule Afadin (AF‐6), encoded by the*MLLT4* gene, is localised at cell‐cell adhesion sites in epithelial cells and fibroblasts to help the formation of adherens junctions (AJs) and maintain cell polarity.^15^, ^16^, ^17^ Originally identified as a fusion partner of the *MLL* gene in acute myeloid leukaemia with chromosome translocation,^18^ AF‐6 is also associated with initiation and progression of solid tumours. Low levels of AF‐6 expression was reported in 15% of breast cancer patients and linked to adverse prognosis,^19^ and the loss of AF‐6 was found to promote pancreatic cancer metastasis by inducing Snail expression,^20^ suggesting that AF‐6 might be a tumour suppressor. In another study, however, elevated AF‐6 expression was closely related to adverse outcomes of breast cancer patients.^21^ Furthermore, phosphorylation of AF‐6 by Akt induced its translocalisation from AJs to the nucleus and increased breast cancer cell migration,^22^ revealing the pro‐tumorigenic role of AF‐6. Therefore, the precise roles of Afadin in tumorigenesis and the underlying mechanisms still remain unclear.

As a classic model organism for the research of developmental biology, *Drosophila* has been recently used in cancer studies and provides great insights into the understanding of tumour initiation and progression.^23^, ^24^ Indeed, the first in vivo evidence for the contributions of polarity proteins to tumorigenesis came from the study of *Drosophila* brains, in which the cells with mutant polarity genes *dlg* or *lgl* displayed overgrown and invasive behaviours.^25^, ^26^ Subsequent studies demonstrated that loss of *scrib*,* dlg* or *lgl* in the larval eye disc accelerated the growth and metastasis of *ras^V12^*‐induced benign tumours, which was largely dependent on the activation of the JNK signalling pathway.^27^, ^28^ In addition to this *ras^V12^*‐polarity defects model, other *Drosophila* models for tumorigenesis have also been developed. For example, co‐overexpression of EGFR and PI3K in larval glia induced neoplasia in *Drosophila* and mimicked glioma.^29^, ^30^ Besides, elevating the levels of the Src kinase in cells along the anterior‐posterior (A/P) compartment boundary of *Drosophila* wing disc produced a metastatic phenotype, providing a model for genetic screening of genes involved in cancer metastasis.^31^, ^32^ Significantly, the core components of the Hippo signalling pathway, which plays vital roles in organ size control and exhibits various mutations in a plethora of tumours, were initially identified using *Drosophila* genetic models, highlighting the reliability of *Drosophila* models for cancer research.^8^, ^33^

In this study, we show that the overexpression of Cno, the *Drosophila* homolog of AF‐6, induces cell proliferation, cell death and cell migration in the larval wing disc. We find that these mixed effects result from strong activation of JNK signalling and Ras‐MAPK signalling. Moderately reducing the activation levels of JNK signalling suppresses the effect of Cno on cell death, thereby inducing massive cell overproliferation and disc overgrowth. In addition, we demonstrate that Hippo signalling acts as a downstream effect or to mediate Cno‐induced proliferation, revealing the underlying mechanism for the pro‐tumorigenic function of Cno in *Drosophila*.

## 2 MATERIALS AND METHODS

### 2.1 Fly stocks

Flies are raised at 25°C under standard conditions. The fly stocks used in this study are as follows: *ptc‐Gal4*,* ap‐Gal4*,* nub‐Gal4*,* gmr‐Gal4, UAS‐GFP/cyo*,* UAS‐GFP/TM6b*,* UAS‐cno‐IR* (NIG 2534R‐3), *UAS‐ras‐IR* (VDRC 106642), *UAS‐mek‐IR* (VDRC 107276). *UAS‐cno* is a gift from Prof. Ulrike Gaul. *UAS‐hep‐IR*,* UAS‐bsk‐IR*,* UAS‐Egr*,* UAS‐Scrib‐IR*,* puc‐lacZ* are gifts from Prof. Lei Xue. *UAS‐yki‐IR*,* ex‐lacZ*,* ban‐lacZ*,* diap1‐lacZ* are gifts from Prof. Lei Zhang.

### 2.2 TUNEL staining

Staining was carried out according to the protocol provided by the manufacturer (MK1021, Boster). Briefly, wing discs from third instar larva were dissected and fixed with 4% formaldehyde in 10 m mol L^−1^ PBS at room temperature for 30 minutes and washed with PBS and distilled water, respectively. Discs were then incubated with labelling buffer in a wet box at 37°C for 2 hours and washed with 0.01 M TBS. After blocking at room temperature for 30 minutes, discs were incubated with anti‐Digoxin at 37°C for 30 minutes. Washed with TBS, discs were reacted with diluted SABC at 37°C for 30 minutes. After washing with TBS, discs were mounted for microscopy.

### 2.3 Immunostaining and image acquisition

Staining was carried out using standard protocols. Briefly, wing discs from third instar larva were dissected and fixed in PBS‐T containing 4% formaldehyde for 20 minutes at room temperature. After washing with PBS‐T, they were blocked with 5% BSA in PBS‐T for 30 minutes and then incubated with primary antibodies overnight at 4°C. Washed discs were subsequently incubated with secondary antibodies. The following primary antibodies were used: mouse anti‐MMP1 (3A6B4, 1:300) and mouse anti‐Wg (4D4, 1:500) were purchased from Developmental Studies Hybridoma Bank. Mouse anti‐β‐galactosidase (sc‐65670, 1:500) was from Santa Cruz. Rabbit anti‐Caspase3 (#9661, 1:1000) and rabbit anti‐p‐Histone H3 (#9701, 1:1000) was from Cell Signaling Technology. Secondary antibodies goat anti‐mouse Alexa Fluor594 (A11012, 1:1000) and goat anti‐rabbit Alexa Fluor594 (A11005, 1:1000) were from Invitrogen. Fluorescence images were recorded using a Nikon DS‐Ri1 fluorescence microscope and a Zeiss LSM 880 confocal microscope. Images of the adult eyes were acquired using a Nikon SMZ‐745T trinocular stereo microscope. Images were then analysed using Zeiss Zen, Image J and Adobe Photoshop software.

## 3 RESULTS

### 3.1 Overexpression of *cno* induces mixed effects in the wing disc

**Figure 1:**
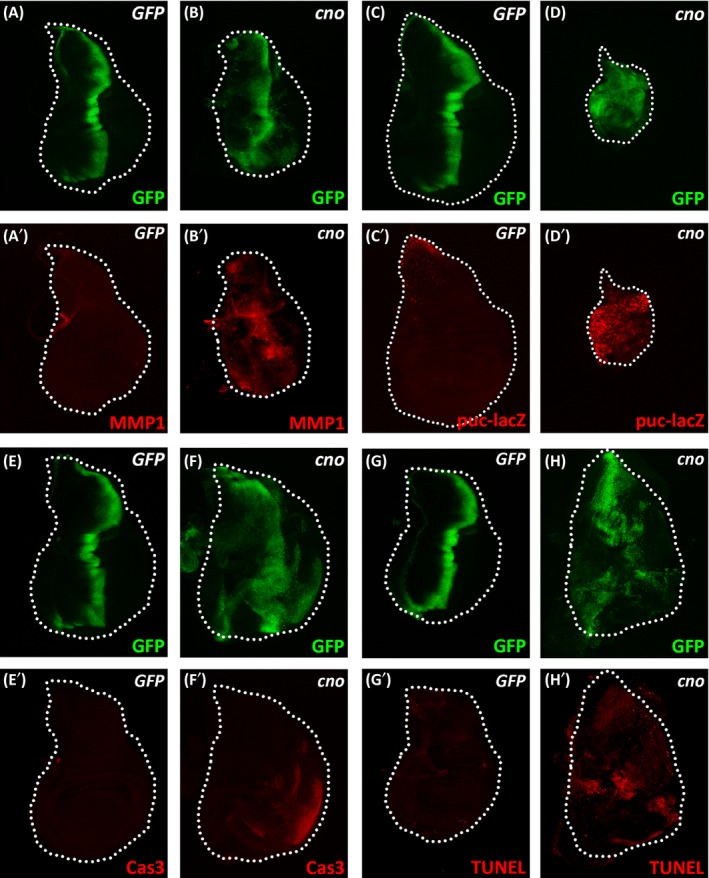
Overexpression of *cno* induces mixed effects. (A‐H) Immnostaining of MMP1, β‐gal, cleaved caspase3, or TUNEL staining in wing discs expressing *ptc‐Gal4 UAS‐GFP*/+; *UAS‐GFP*/+ (A, E and G), *ptc‐Gal4 UAS‐GFP*/+; *UAS‐cno*/+ (B, F and H), *ptc‐Gal4 UAS‐GFP*/+; *UAS‐GFP*/*puc‐lacZ* (C) or *ptc‐Gal4 UAS‐GFP*/+; *UAS‐cno*/*puc‐lacZ* (D). White dashed lines indicate the edges of the wing discs. Note the reduced disc size in the presence of enhancer‐trapped *puc‐lacZ*, which additionally increases JNK activity due to the removal of one copy of *puc*

To explore the role of *Drosophila* AF‐6 in cell proliferation, we overexpressed *cno* at the A/P compartment boundary of *Drosophila* wing disc by *ptc‐Gal4*. The expression domain of *cno*, labelled by GFP‐positive cells, became broader and irregular than the control, with some cells migrating into the posterior compartment of the disc (Figure 1A,B, Supporting Information Figure S1D). We monitored the signal of the mitosis marker, phosphorylated Histone H3 (pH3) and observed a significant increase in the number of pH3‐positive cells in the region of cno overexpression in the wing disc (Supporting Information Figure S1 A‐C). The activation of JNK signalling has a well‐established role in inducing cell migration in *Drosophila* wing disc.^34^, ^35^, ^36^ We therefore examined the expression of JNK target genes in these wing discs. Significantly elevated expression of JNK targets, including the matrix metalloproteinase *MMP1* and an enhancer‐trapped lacZ reporter of the JNK inhibitor *puckered* (*puc*), was observed in *cno* expressing domain (Figure 1A‐D). The activation of JNK signalling can either promote cell proliferation or trigger cell death, depending on the levels of activation. Whereas, mild to moderate JNK activation is linked to proliferation, strong JNK activation additionally causes cell death.^37^, ^38^, ^39^, ^40^ Indeed, we observed increased cell death in the wing discs overexpressing *cno*, as indicated by the immunostaining of cleaved caspase3 and TUNEL staining (Figure 1E‐H). Thus, overexpression of *cno* in *Drosophila* wing disc induces mixed effects on cell proliferation, cell death and cell migration, which are connected to strong activation of JNK signalling.

### 3.2 Moderate alleviation of JNK activation augments Cno‐mediated proliferation

**Figure 2:**
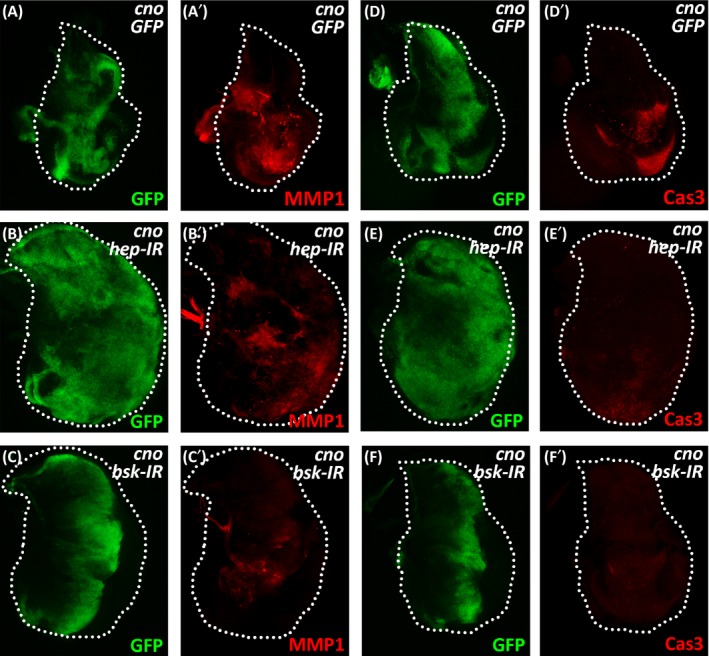
The levels of JNK activation on Cno‐induced proliferation. (A‐F) Immunostaining of MMP1 and cleaved caspase3 in wing discs expressing *ptc‐Gal4 UAS‐GFP*/*UAS‐GFP*;* UAS‐cno*/+ (A and D), *ptc‐Gal4 UAS‐GFP*/+; *UAS‐cno*/*UAS‐hep‐IR* (B and E) or *ptc‐Gal4 UAS‐GFP*/+; *UAS‐cno*/*UAS‐bsk‐IR* (C and F). White dashed lines indicate the edges of the wing discs

To investigate the roles of the JNK pathway in Cno‐mediated phenotypes in *Drosophila* wing disc, we examined the effects of differentially reducing the levels of Cno‐induced JNK activation. *hemipterous* (*hep*) and *basket* (*bsk*) encode the *Drosophila* homolog of JNKK and JNK, respectively. The knockdown of *hep* or *bsk* displayed different levels of suppression on activated JNK signalling (Supporting Information Figure S2). In a *scrib* knockdown induced mild JNK activation model, both *hep* knockdown and *bsk* knockdown effectively suppressed the activated expression of *MMP1* (Supporting Information Figure S2A‐D). In contrast, in an *eiger* (*egr*, the *Drosophila* tumour necrosis factor superfamily ligand) overexpression‐induced small eye model, the knockdown of *hep* only weakly recovered the size of the eye, whereas, the knockdown of *bsk* largely rescued the eye phenotype (Supporting Information Figure S2E‐H). These data indicate that the knockdown of *hep* moderately inhibits the activation of JNK signalling, while the knockdown of *bsk* inhibits it more effectively. As suggested by the expression domain of GFP, the knockdown of *hep* or *bsk* at the A/P compartment boundary had no effect on cell proliferation or migration (Supporting Information Figure S3). Interestingly, *cno* overexpression in the presence of *hep* knockdown at the A/P compartment boundary induced massive cell proliferation and disc overgrowth, with the GFP‐positive cells spreading throughout the wing disc (Figure 2A,B, Supporting Information Figure S4). The combination with *bsk* knockdown displayed similar but weaker phenotype (Figure 2C). Similar effects by the knockdown of *hep* or *bsk* were also observed when *cno* was overexpressed in other compartments of the wing disc (Supporting Information Figure S5). The induction of *MMP1* expression by *cno* overexpression was weakened by *bsk* knockdown but barely affected by *hep* knockdown, suggesting the requirement for the activation of JNK signalling to mediate the function of Cno in massive cell proliferation and migration (Figure 2A‐C). In addition, either *hep* knockdown or *bsk* knockdown largely eliminated the cell apoptosis induced by *cno* overexpression (Figure 2D‐F). Therefore, moderate alleviation of Cno‐induced JNK activation by *hep* knockdown predominantly displays growth‐promoting and pro‐migration effects, which can serve as a new type of *Drosophila* tumour model. In contrast, strong inhibition of Cno‐induced JNK activation by *bsk* knockdown only moderately promotes cell proliferation and migration. Importantly, the knockdown of *cno* in the presence of *hep* knockdown did not stimulate cell proliferation or migration, indicating that our observations above were not caused by dominant negative effects (Supporting Information Figure S6).

### 3.3 Cno induces proliferation via the Hippo signalling pathway

**Figure 3:**
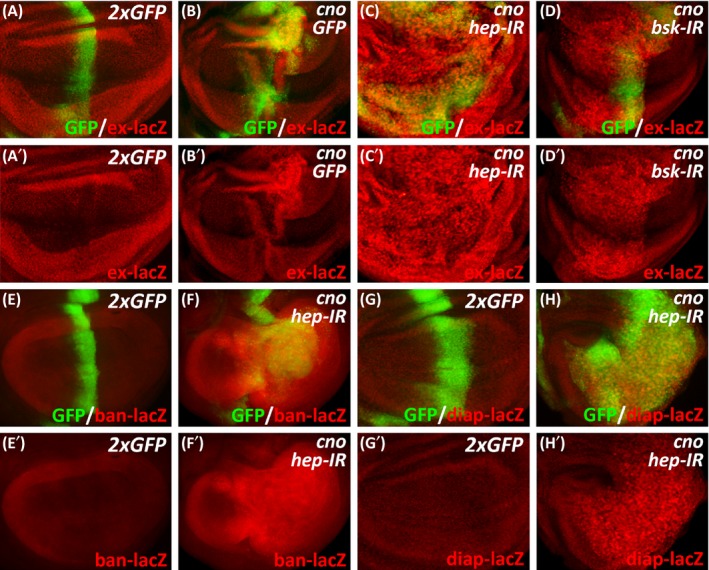
Cno‐mediated overgrowth depends on the Hippo signalling pathway. (A‐H) β‐galstaining of wing discs expressing *ptc‐Gal4 UAS‐GFP*/*ex‐lacZ*;* UAS‐GFP*/*UAS‐GFP* (A), *ptc‐Gal4 UAS‐GFP*/*ex‐lacZ*;* UAS‐cno*/*UAS‐GFP* (B), *ptc‐Gal4 UAS‐GFP*/*ex‐lacZ*;* UAS‐cno*/*UAS‐hep‐IR* (C), *ptc‐Gal4 UAS‐GFP*/*ex‐lacZ*;* UAS‐cno*/*UAS‐bsk‐IR* (D), *ptc‐Gal4 UAS‐GFP*/*ban‐lacZ*;* UAS‐GFP*/*UAS‐GFP* (E), *ptc‐Gal4 UAS‐GFP*/*ban‐lacZ*;* UAS‐cno*/*UAS‐hep‐IR* (F), *ptc‐Gal4 UAS‐GFP*/*UAS‐GFP*;* UAS‐GFP*/*diap‐lacZ* (G) or *ptc‐Gal4 UAS‐GFP*/+; *UAS‐cno UAS‐hep‐IR*/*diap‐lacZ* (H)

**Figure 4:**
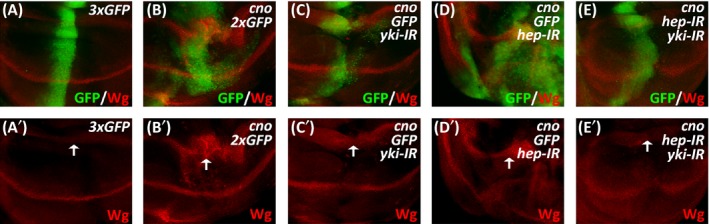
The effects of *yki* knockdown on Cno‐mediated overgrowth. (A‐E) Immunostaining of Wg in wing discs expressing *ptc‐Gal4 UAS‐GFP*/*UAS‐GFP*;* UAS‐GFP*/*UAS‐GFP* (A), *ptc‐Gal4 UAS‐GFP*/*UAS‐GFP*;* UAS‐cno*/*UAS‐GFP* (B), *ptc‐Gal4 UAS‐GFP*/*UAS‐yki‐IR*;* UAS‐cno*/*UAS‐GFP* (C), *ptc‐Gal4 UAS‐GFP*/*UAS‐GFP*;* UAS‐cno*/*UAS‐hep‐IR* (D) or *ptc‐Gal4 UAS‐GFP*/*UAS‐yki‐IR*;* UAS‐cno*/*UAS‐hep‐IR* (E). White arrows indicate Wg signals at the hinge region of the wing discs

The Hippo signalling pathway is an evolutionarily conserved signal transduction pathway that plays vital roles in cell proliferation and apoptosis to control cell fate, organ size and tissue homeostasis.^8^ It has been reported that multiple components of the Hippo pathway are regulated directly by the cell adhesion and polarity machinery, and defects in cell polarity may result in tumorigenesis via dysfunction of the Hippo pathway.^41^ To investigate whether Cno‐induced overgrowth is mediated by the Hippo pathway, we examined the expression of target genes of Yorkie (Yki), the key transcriptional coactivator of Hippo signalling. We observed that the expression of Yki target gene *expanded* (*ex*) was prominently activated upon the overexpression of *cno*, which was further enhanced in the presence of *hep* knockdown or *bsk* knockdown (Figure 3A‐D). Noticeably, the knockdown of *bsk* was less potent in enhancing the expression of *ex* than the knockdown of *hep*, revealing the requirement of JNK activation for the induction of Yki targets (Figure 3C,D). Similarly, activated expression of other Yki target genes such as *bantam* (*ban*) and *diap1* was also observed in this tumour model (Figure 3E‐H). Importantly, the knockdown of *yki* greatly reduced Cno‐induced cell overproliferation, even in the presence of *hep* knockdown (Figure 4). Consistently, Cno‐induced expression of Yki target gene *wingless* (*wg*) was largely suppressed by the knockdown of *yki* (Figure 4). These results confirm that Cno‐induced cell overproliferation depends on Hippo signalling and requires the induction of Yki target gene expression.

### 3.4 The Ras‐MAPK signalling pathway regulates Cno‐induced cell proliferation

**Figure 5:**
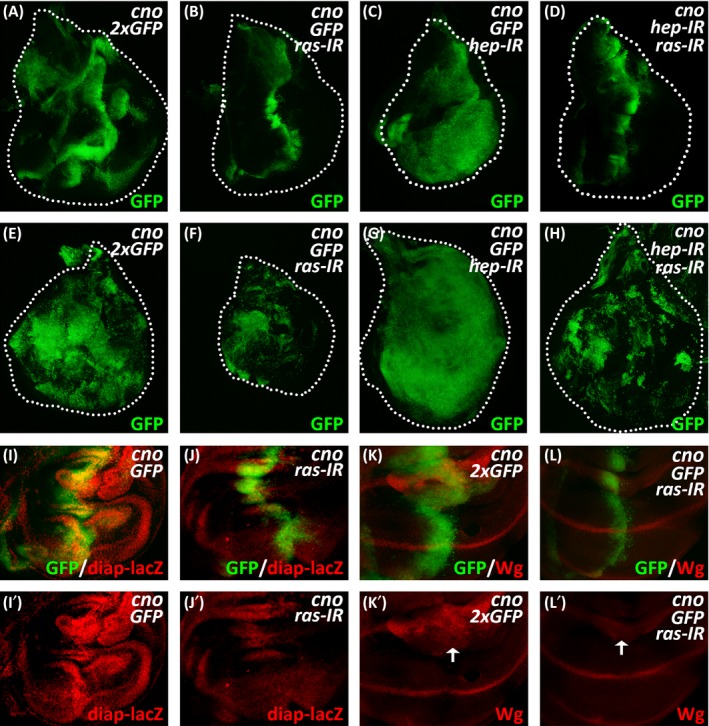
Ras‐MAPK signalling acts between Cno and Hippo signalling. (A‐D) The proliferation of GFP‐positive cells at the A/P compartment boundary of the wing disc expressing *ptc‐Gal4 UAS‐GFP*/*UAS‐GFP*;* UAS‐cno*/*UAS‐GFP* (A), *ptc‐Gal4 UAS‐GFP*/*UAS‐ras‐IR*;* UAS‐cno*/*UAS‐GFP* (B), *ptc‐Gal4 UAS‐GFP*/*UAS‐GFP*;* UAS‐cno*/*UAS‐hep‐IR* (C) or *ptc‐Gal4 UAS‐GFP*/*UAS‐ras‐IR*;* UAS‐cno*/*UAS‐hep‐IR* (D). (E‐H) The proliferation of GFP‐positive cells in the dorsal region of the wing discs expressing *ap‐Gal4 UAS‐GFP*/*UAS‐GFP*;* UAS‐cno*/*UAS‐GFP* (E), *ap‐Gal4UAS‐GFP*/*UAS‐ras‐IR*;* UAS‐cno*/*UAS‐GFP*(F), *ap‐Gal4 UAS‐GFP*/*UAS‐GFP*;* UAS‐cno*/*UAS‐hep‐IR* (G) or *ap‐Gal4 UAS‐GFP*/*UAS‐ras‐IR*;* UAS‐cno*/*UAS‐hep‐IR* (H). White dashed lines display the edges of the wing discs. (I‐L) Imunnostaining of β‐gal or Wg in wing disc expressing *ptc‐Gal4 UAS‐GFP*/*UAS‐GFP*;* UAS‐cno*/*diap‐lacZ* (I), *ptc‐Gal4 UAS‐GFP*/*UAS‐ras‐IR*;* UAS‐cno*/*diap‐lacZ* (J), *ptc‐Gal4 UAS‐GFP*/*UAS‐GFP*;* UAS‐cno*/*UAS‐GFP* (K) or *ptc‐Gal4 UAS‐GFP*/*UAS‐ras‐IR*;* UAS‐cno*/*UAS‐GFP* (L). White arrows indicate Wg signals at the hinge region of the wing discs

As strong inhibition of JNK activation still effectively activated the expression of Yki target genes, we speculated that Cno might affect additional signalling, in addition to JNK signalling, to regulate the Hippo pathway. The Ras‐MEK‐ERK pathway is one branch of the Mitogen‐activated protein kinase (MAPK) pathways. It transduces extracellular signals into the nucleus through sequential activation of the MEKK‐MEK‐MAPK kinase cascade, regulates multiple physiological processes such as cell proliferation, differentiation and migration, and participates in the regulation of Hippo signalling.^34^, ^42^, ^43^, ^44^, ^45^ To investigate whether the Ras‐MAPK pathway is involved in Cno‐induced overproliferation, we examined the effects of *ras* knockdown in above genetic setups. As controls, the knockdown of *ras* at the A/P compartment boundary or the dorsal compartment of the wing disc showed no effect on cell proliferation (Supporting Information Figure S7). In contrast, cell overproliferation induced by the overexpression of *cno*, in the absence or presence of *hep* knockdown, was strongly inhibited by the knockdown of *ras* (Figure 5A‐H). Intriguingly, despite the effective inhibition of cell overproliferation, the knockdown of *ras* seemed unable to block cell migration induced by *cno* overexpression as was achieved by *bsk* knockdown (Figure 2C and Figure 5A‐H). These data suggest that Cno‐induced phenotypes can be partially separated and are mediated by distinct pathways. Whereas, the Ras‐MAPK pathway specifically contributes to the regulation of cell proliferation, the JNK pathway regulates both cell proliferation and migration. It has been reported that the Ras‐MAPK signalling influences cell growth through an Yki‐dependent mechanism.^46^ We thus examined whether the effects of *ras* knockdown on Cno‐induced proliferation were dependent on the Hippo pathway. Indeed, the knockdown of *ras* significantly impaired Cno‐induced expression of Yki target genes (*diap1* and *wg*) in the wing disc (Figure 5I‐L). In addition, the knockdown of *MEK*, another core component of Ras‐MAPK signalling, exhibited similar effects as the knockdown of *ras* on Cno‐induced phenotypes in the wing disc, including cell proliferation, cell migration and Yki target gene expression (Supporting Information Figure S8). Together, our results suggest that Cno can activate both JNK and Ras‐MAPK signalling and regulate cell proliferation via the downstream Hippo signalling.

## 4 DISCUSSION

Although dual regulation of cell proliferation by polarity proteins have been observed and studied for quite a period, controversies about the precise roles of cell polarity in such an important cellular process still exist.^13^ For instance, the adhesion molecular Afadin, which contributes to forming and sustaining cellular junctions and cell polarity, has been reported to have pro‐ or anti‐tumorigenic functions.^47^ In this work, by expressing Cno, the *Drosophila* homolog of Afadin, we observed mixed phenotypes of cell death, growth and migration. Since the JNK signalling pathway is well known to participate in all of these cellular processes,^34^ we thus checked the activity of the JNK pathway and indeed found elevated expression of the JNK target genes. However, when we attempted to dissect the mixed phenotypes by knocking down key kinases of the JNK pathway, we observed unexpected outcomes. Particularly, knocking down *hep* greatly promoted Cno‐induced overgrowth, while knocking down *bsk* had a milder effect. Given the stronger inhibition of the JNK activation by the knockdown of *bsk* than the knockdown of *hep*, we conclude that moderately alleviating the levels of JNK activation can largely assist Cno‐mediated cell proliferation.

Strong activation of the JNK pathway mainly triggers cell death and migration instead of the mixture of cell death and proliferation, implying that additional mechanisms beyond the JNK pathway underlie the effects of*cno* overexpression. Since the Hippo signalling pathway is one of the most well‐known regulators that control the process of cell proliferation and is frequently associated with tumour initiation and progression,^8^, ^41^ we also examined the downstream readouts of this pathway in wing disc with *cno* overexpression, and confirmed the involvement of the Hippo pathway. Essentially, knocking down *hep* substantially increased the expression of Hippo target genes induced by *cno* overexpression, while knocking down *bsk* exerted lesser effects on them, suggesting that moderate activation of the JNK pathway also promotes cell growth via the regulation of Hippo signalling.

It is proposed that the contribution of the JNK signalling to cell proliferation is converted from anti‐ to pro‐tumorigenic by Ras,^48^ the core component of the Ras‐MAPK signalling pathway. We thus investigated the roles of the Ras‐MAPK pathway in Cno‐mediated phenotypes. We found that the knockdown of either *ras* or its downstream kinase MEK significantly inhibited Cno‐mediated cell proliferation, especially in the context of the massive overgrowth induced by concurrent *hep* knockdown. Notably, neither ras nor *mek* knockdown was capable of reducing Cno‐induced cell death and migration, implying that there exist multiple mechanisms that are responsible for the mixed phenotypes induced by *cno* overexpression. Importantly, by examining the expression of several Hippo targets, we could determine that the effects of *ras* or *mek* knockdown were mediated by the regulation of Hippo signalling.

**Figure 6:**
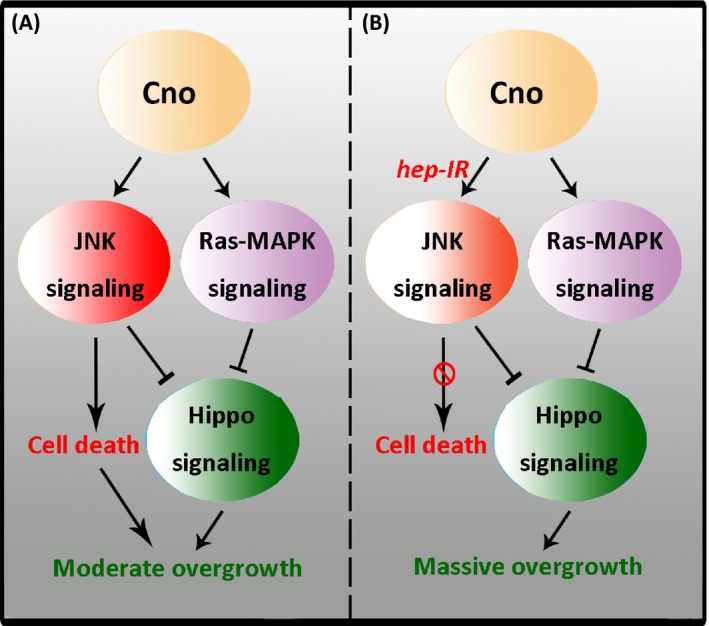
The working model for Cno‐mediated effects on cell proliferation. A, Overexpression of *cno* simultaneously activates JNK and Ras‐MAPK signalling, inducing both cell proliferation and cell death. B, Moderately reducing the activation levels of JNK signalling suppresses the effect of Cno on cell death, inducing massive cell overproliferation via Hippo signalling

Based on the observations above, we propose a model for the mechanisms underlying Cno‐mediated phenotypes (Figure 6). Overexpression of *cno* in *Drosophila* wing disc activates both the JNK and Ras‐MAPK signalling pathways. While the Ras‐MAPK pathway mainly promotes cell growth via Hippo signalling, the effects of the JNK pathway are more complicated. When the JNK pathway is strongly activated by *cno* overexpression, it predominantly triggers cell death and cell migration. Combined with the overgrowth induced by the Ras‐Hippo axis, the cells display mixed phenotypes of death and growth. However, when the levels of JNK activation are moderately reduced, it mostly promotes cell growth rather than cell death via the Hippo pathway. Under this context, cooperating with the Ras‐MAPK signalling, the cells display massive overproliferation.

In conclusion, the influences of polarity proteins on cell fate determination are complicated and context‐dependent in many occasions. As illustrated in this study, the polarity protein Cno activates both the JNK pathway and the Ras‐MAPK pathway. Importantly, polarity proteins affect cell fate not only by disruption of cell polarity, but also by acting as signal transducing molecules to participate in signalling pathways that regulate cell death and proliferation. Therefore, the use of anti‐cancer drugs or treatment targeting polarity proteins must consider their complicated roles in cell proliferation and the signalling pathways they are associated with. In addition, the combination of *cno* expression and *hep* knockdown in *Drosophila* wing disc provides a new model for studying cell proliferation and tumour formation in this classic model organism.

## CONFLICT OF INTEREST

The authors declare no conflict of interest.

## AUTHOR CONTRIBUTIONS

HS designed the research; ZM, PL and XH performed the experiments; HS and ZM analysed the data and wrote the paper.
