# Supplementary material for: Polarity protein Canoe mediates overproliferation via modulation of JNK, Ras‐MAPK and Hippo signalling
Source: Cell Prolif. 2018 Oct 17;52(1):e12529. doi: 10.1111/cpr.12529 (PMC6430484; doi:10.1111/cpr.12529)
Supplement: Supplementary file 1 [file CPR-52-e12529-s001.doc]

**Supplementary Information**


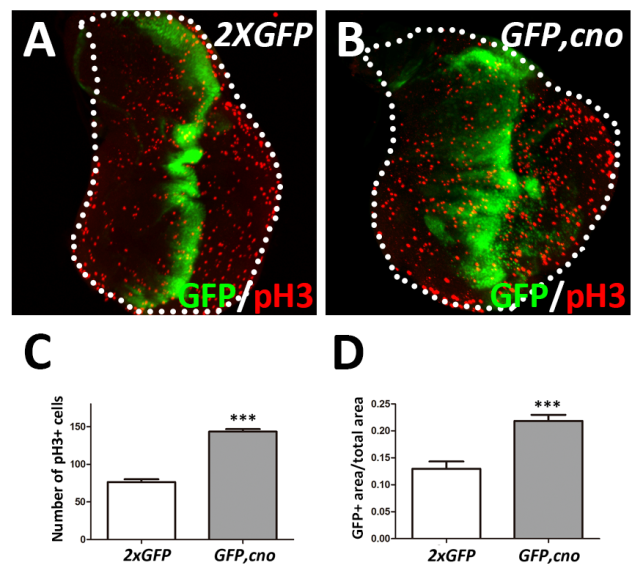


**Figure S1 The effects of *cno* overexpression on cell proliferation.** (A and B) Immunotaining of pH3 in wing discs expessing *ptc-Gal4 UAS-GFP*/*+*; *UAS-GFP*/*UAS-GFP* (A) or *ptc-Gal4 UAS-GFP*/*UAS-GFP*; *UAS-cno*/*+* (B). (C) Quantification of the number of pH3-positive cells at the A/P boundary labeled with GFP. n=4 for each group. *** p<0.001, students’ t test. (D) Quantification of the ratio of GFP-positive area to total area of the wing disc. n=7 for each group. *** p<0.001, students’ t test.


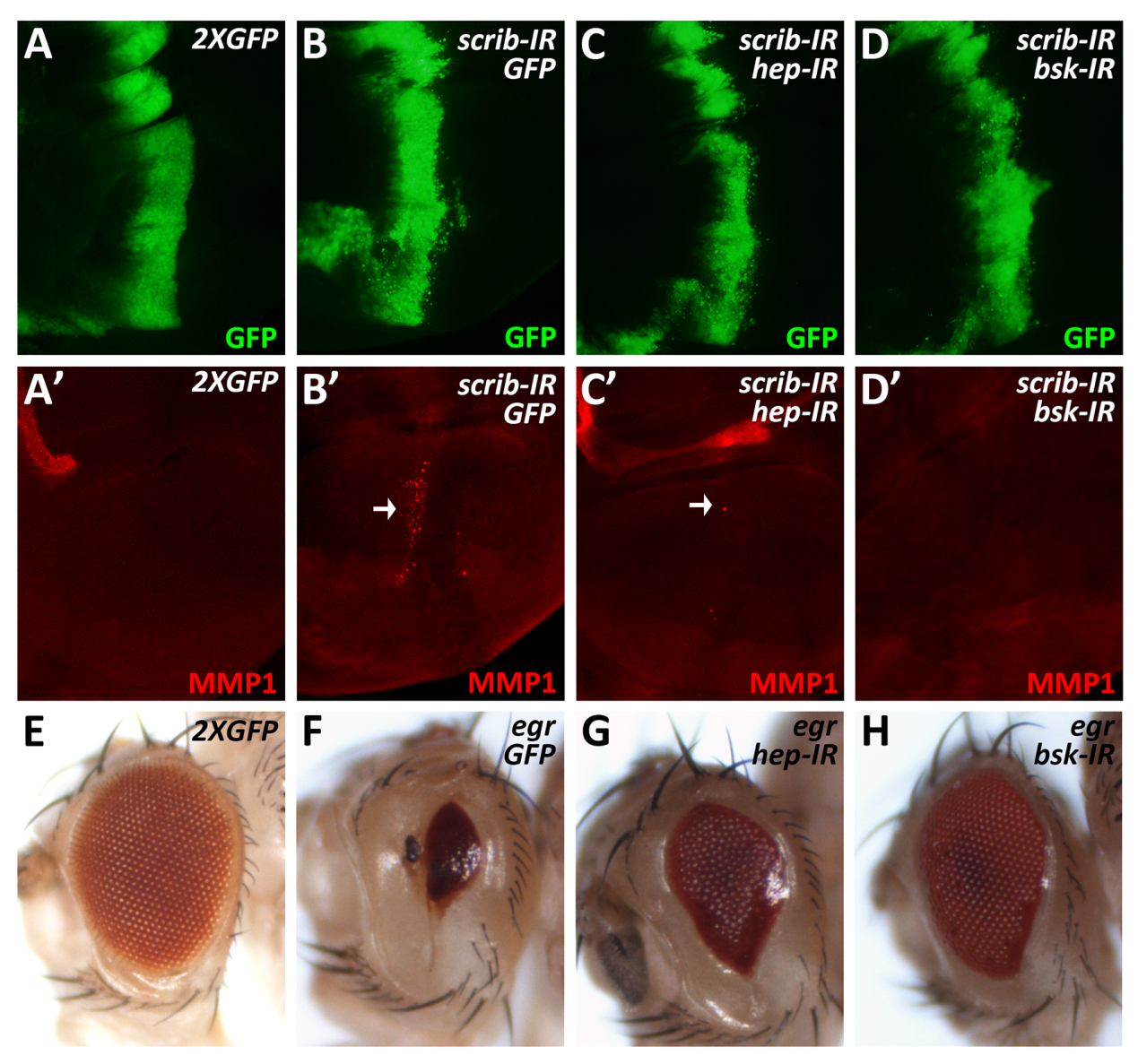


**Figure S2 The effects of reducing the dosage of *hep* or *bsk* on the activity of JNK signaling.** (A-D) Immunotaining of MMP1 in wing discs expressing *ptc-Gal4 UAS-GFP*/+; *UAS-GFP*/*UAS-GFP* (A), *ptc-Gal4 UAS-GFP UAS-scrib-IR*/+; *UAS-GFP*/+ (B), *ptc-Gal4 UAS-GFP UAS-scrib-IR*/+; *UAS-hep-IR*/+ (C), *ptc-Gal4 UAS-GFP UAS-Scrib-IR*/+; *UAS-bsk-IR*/+ (D). Arrows indicate signals of MMP1 staining. (E-H) Images of adult eyes expressing *UAS-GFP*/+; *gmr-Gal4*/*UAS-GFP* (E), *UAS-egr*/+; *gmr-Gal4*/*UAS-GFP* (F), *UAS-egr*/+; *gmr-Gal4*/*UAS-hep-IR* (G), *UAS-egr*/+; *gmr-Gal4*/*UAS-bsk-IR* (H).


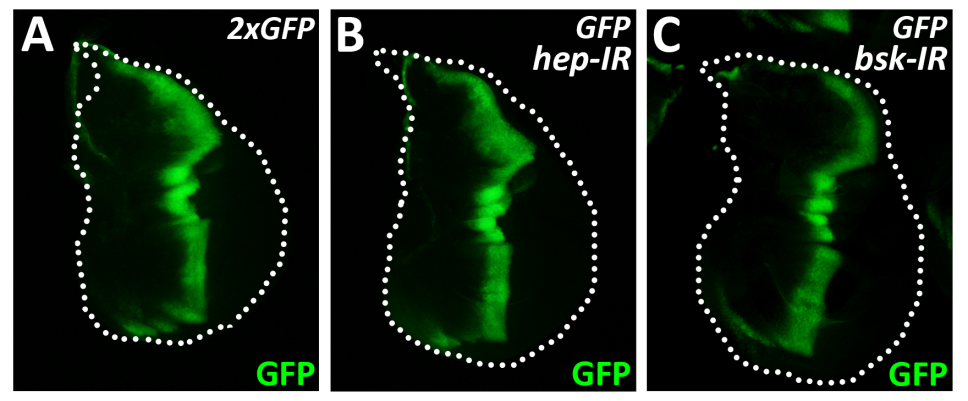


**Figure S3 The effects of reducing the activity of JNK signaling on cell proliferation.** (A-C) The effects of knocking down *hep* or *bsk* on cell proliferation at the A/P boundary of wing discs expressing *ptc-Gal4 UAS-GFP*/+; *UAS-GFP*/*UAS-GFP* (A), *ptc-Gal4 UAS-GFP*/+; *UAS-GFP*/*UAS-hep-IR* (B) or *ptc-Gal4 UAS-GFP*/+; *UAS-GFP*/*UAS-bsk-IR* (C). White dashed lines display the edges of the wing discs.


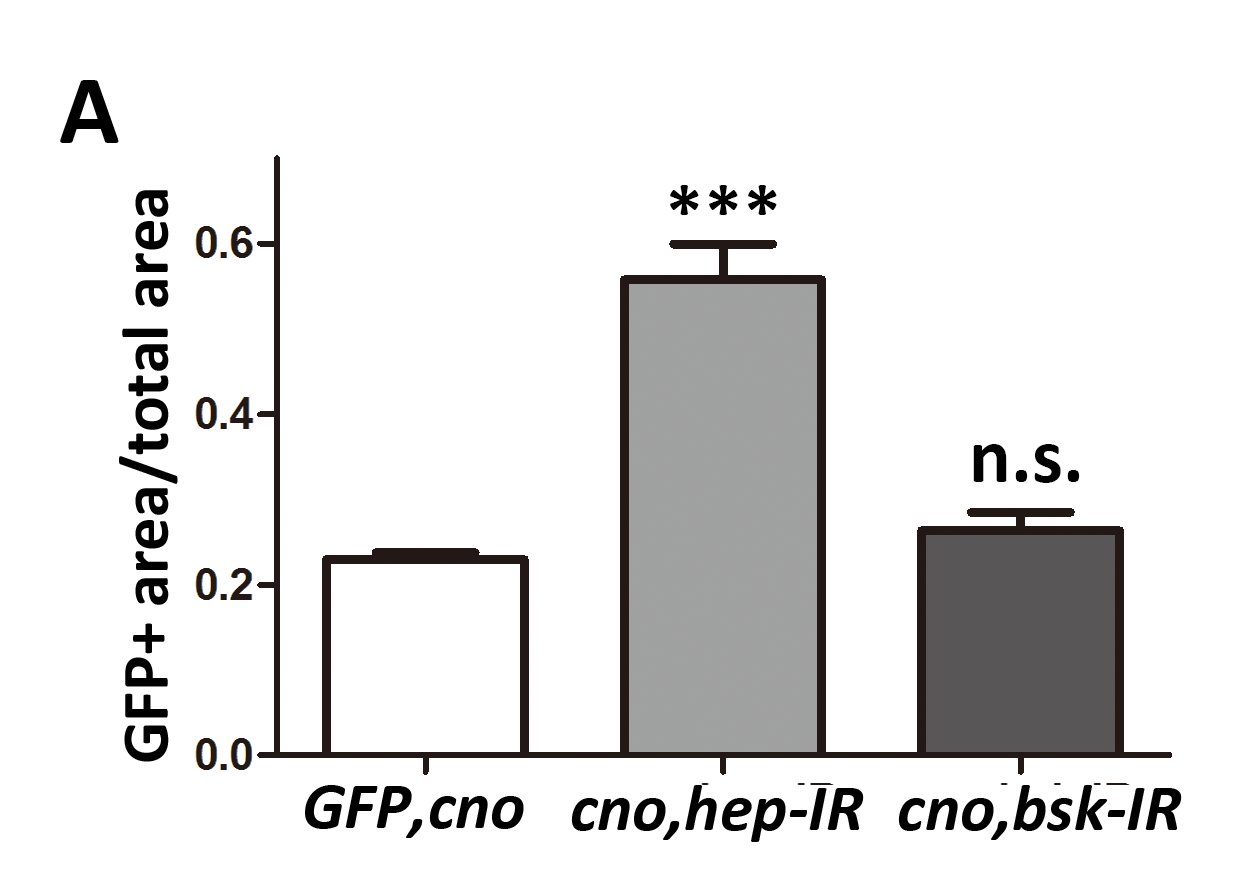


**Figure S4 Quantification of the effects of JNK inhibition on Cno-induced proliferation.** (A) Quantification of the ratio of GFP-positive area to total area of the wing disc. n=6. *** p<0.001, n.s. p=0.09, students’ t test.


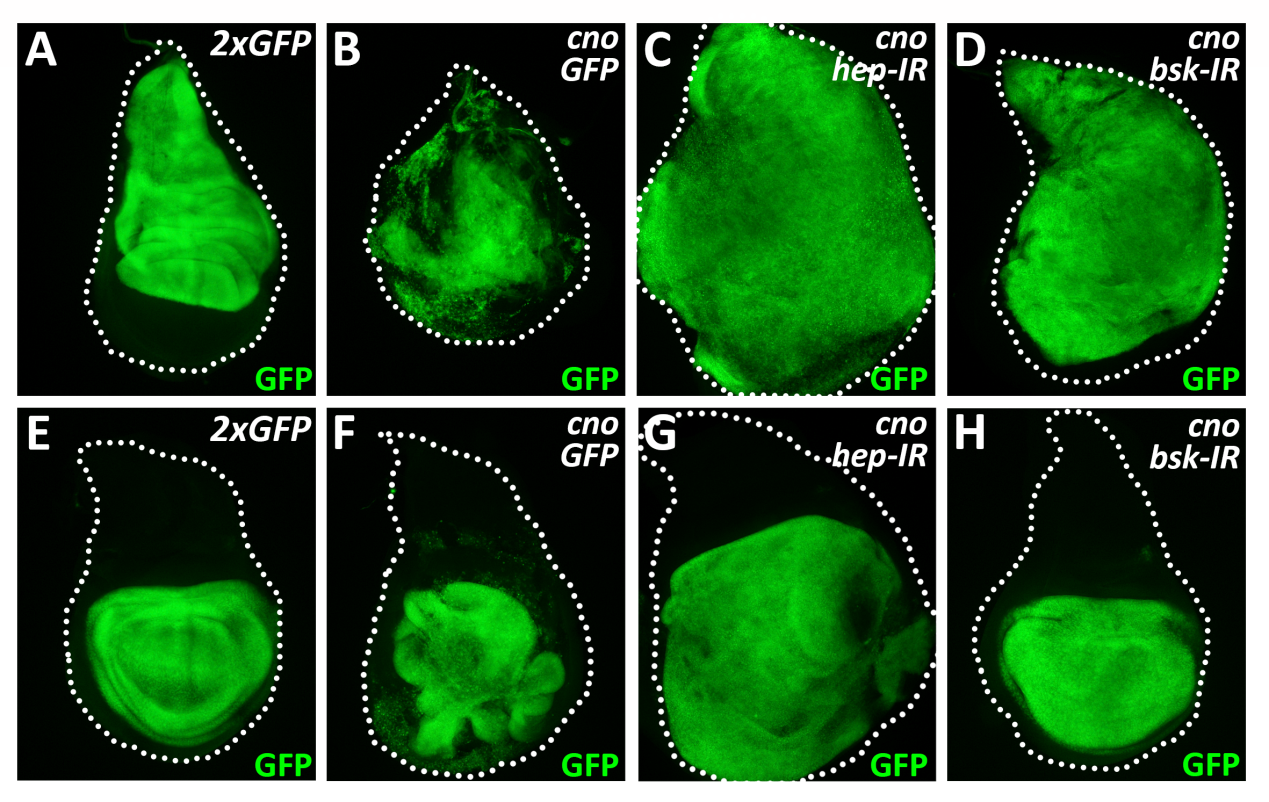


**Figure S5 The levels of JNK activation on Cno-mediated cell proliferation.** (A-D) The proliferation of GFP-positive cells in the dorsal region of wing discs expressing *ap-Gal4 UAS-GFP*/+; *UAS-GFP*/*UAS-GFP* (A), *ap-Gal4 UAS-GFP*/*UAS-GFP*; *UAS-cno*/+ (B), *ap-Gal4 UAS-GFP*/+; *UAS-cno*/*UAS-hep-IR* (C) or *ap-Gal4 UAS-GFP*/*UAS-bsk-IR; UAS-cno*/+ (D). (E-H) The proliferation of GFP-positive cells in the pouch region of wing discs expressing *nub-Gal4*/*UAS-GFP*; *UAS-GFP*/*UAS-GFP* (E), *nub-Gal4*/+; *UAS-cno UAS-GFP*/*UAS-GFP* (F), *nub-Gal4*/+; *UAS-cno UAS-hep-IR*/*UAS-GFP* (G) or *nub-Gal4*/*UAS-bsk-IR*; *UAS-cno*/*UAS-GFP* (H).White dashed lines display the edges of the wing discs.


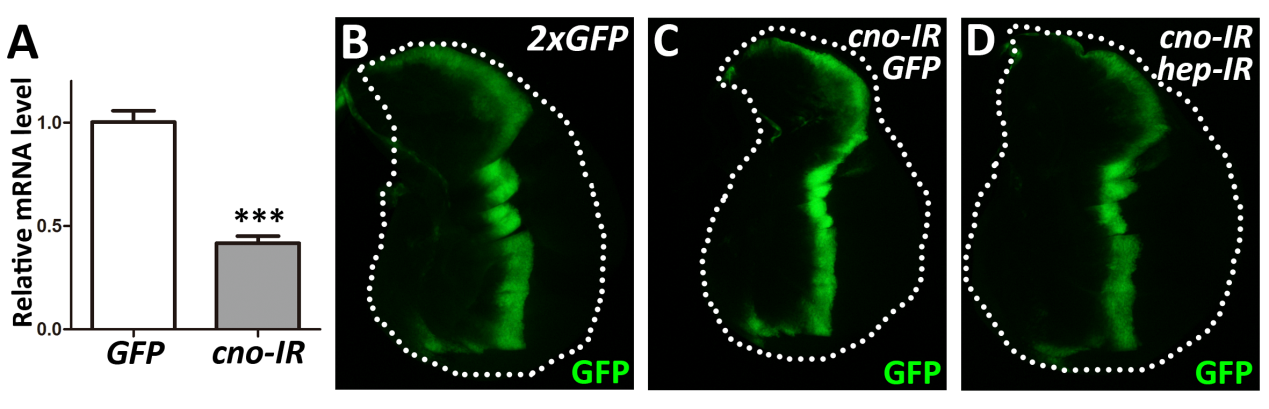


**Figure S6 The effects of reducing the dosage of *cno* on cell proliferation.** (A) The knockdown efficiency of *cno-IR*. ***, *P*<0.001, Student’s *t*-test (n=3). (B-D) The effects of knocking down *cno* with or without *hep* knockdown on cell proliferation at the A/P boundary of wing discs expressing *ptc-Gal4 UAS-GFP*/+; *UAS-GFP*/*UAS-GFP* (B), *ptc-Gal4 UAS-GFP*/+; *UAS-cno-IR*/*UAS-GFP* (C) or *ptc-Gal4 UAS-GFP*/+; *UAS-cno-IR*/*UAS-hep-IR* (D). White dashed lines display the edges of the wing discs.


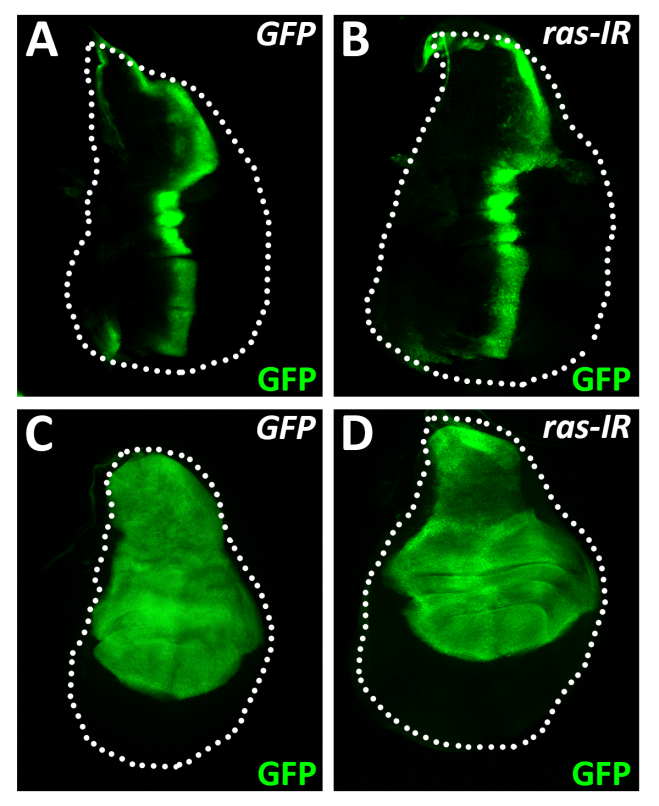


**Figure S7 The effects of reducing the dosage of *ras* on cell proliferation.** (A and B) Wing discs expressing *ptc-Gal4 UAS-GFP*/+; *UAS-GFP/+* (A) or *ptc-Gal4 UAS-GFP*/*UAS-ras-IR* (B). **(**C and D) Wing discs expressing*ap-Gal4 UAS-GFP*/*+; UAS-GFP/+* (C), *ap-Gal4 UAS-GFP*/*UAS-ras-IR* (D). White dashed lines display the edges of the wing discs.


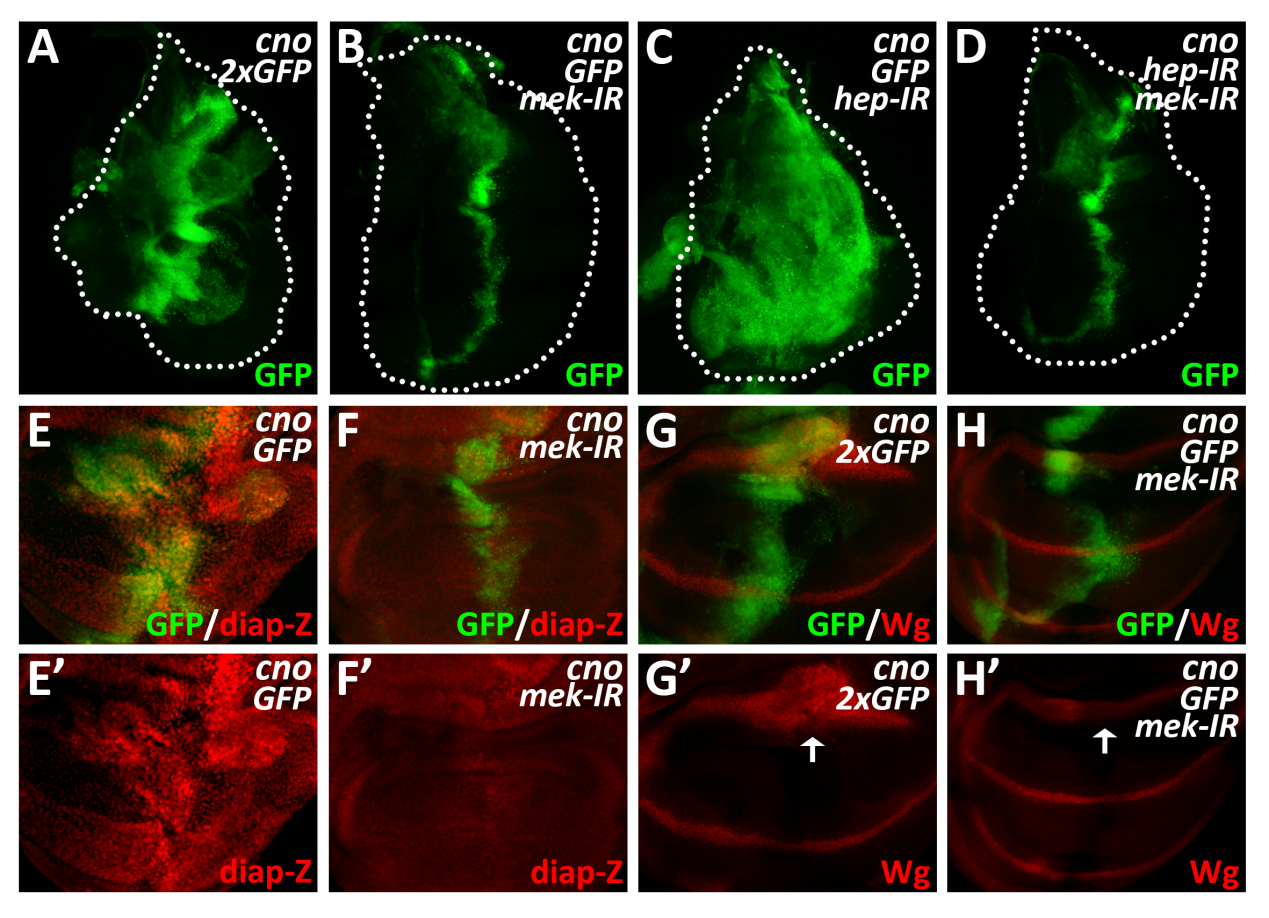


**Figure S8 MEK mediates Cno-induced overgrowth via Hippo signaling.** (A-D) The proliferation of cells at the A/P boundary of wing discs expressing *ptc-Gal4 UAS-GFP*/*UAS-GFP*; *UAS-cno*/UAS-GFP (A), *ptc-Gal4 UAS-GFP*/*UAS-mek-IR*; *UAS-cno*/*UAS-GFP* (B), *ptc-Gal4 UAS-GFP*/*UAS-GFP*; *UAS-cno*/*UAS-hep-IR* (C) or *ptc-Gal4 UAS-GFP*/*UAS-mek-IR*; *UAS-cno*/*UAS-hep-IR* (D). White dashed lines display the edges of the wing discs. (E-H) Imunnostaining of β-gal or Wg in wing discs expressing *ptc-Gal4 UAS-GFP*/*UAS-GFP*; *UAS-cno*/*diap-lacZ* (E), *ptc-Gal4 UAS-GFP*/*UAS-mek-IR*; *UAS-cno*/*diap-lacZ* (F), *ptc-Gal4 UAS-GFP*/*UAS-GFP*; *UAS-cno*/*UAS-GFP* (G) or *ptc-Gal4 UAS-GFP*/*UAS-mek-IR*; *UAS-cno*/*UAS-GFP* (H). White arrows indicate Wg signals at the hinge region of the wing discs.
